# Physicochemical investigation of shrimp fossils from the Romualdo and Ipubi formations (Araripe Basin)

**DOI:** 10.7717/peerj.6323

**Published:** 2019-02-14

**Authors:** Olga Alcântara Barros, João Hermínio Silva, Gilberto Dantas Saraiva, Bartolomeu Cruz Viana, Alexandre Rocha Paschoal, Paulo Tarso Cavalcante Freire, Naiara Cipriano Oliveira, Amauri Jardim Paula, Maria Somália Viana

**Affiliations:** 1 Department of Geology, Universidade Federal do Ceará, Fortaleza, Ceará, Brazil; 2 Campus Juazeiro do Norte, Universidade Federal do Cariri, Juazeiro do Norte, Ceará, Brazil; 3 Faculdade de Educação, Ciências e Letras do Sertão Central, Universidade Estadual do Ceará, Quixadá, Ceará, Brazil; 4 Department of Physics, Universidade Federal do Piauí, Teresina, Piauí, Brazil; 5 Department of Physics, Universidade Federal do Ceará, Fortaleza, Ceará, Brazil; 6 Laboratório de Paleontologia, Museu Dom José, Universidade Estadual do Vale do Acaraú, Sobral, Ceará, Brazil

**Keywords:** Shrimp fossil, Energy-Dispersive X-Ray Spetroscopy, Vibrational spectroscopy

## Abstract

The Ipubi and Romualdo Formations are Cretaceous units of the Araripe Basin (Santana Group). The first and most ancient was deposited in a lake environment, and some fossils were preserved in shales deposited under blackish conditions. The second was deposited in a marine environment, preserving a rich paleontological content in calcareous concretions. Considering that these two environments preserved their fossils under different processes, in this work we investigated the chemical composition of two fossilized specimens, one from each of the studied stratigraphic units, and compared them using vibrational spectroscopy techniques (Raman and IR), X-ray diffraction and large-field energy-dispersive X-ray spectroscopy (EDS) mappings. Calcite was observed as the dominant phase and carbon was observed in the fossils as a byproduct of the decomposition. The preservation of hydroxide calcium phosphate (Ca_10_(PO_4_)_6_(OH)_2_, hydroxyapatite) was observed in both fossils. In addition, it was observed that there was a smaller amount of pyrite (pyritization) in the Romualdo Formation sample than in the Ipubi one. Large-field EDS measurements showed the major presence of the chemical elements calcium, oxygen, iron, aluminum and fluoride in the Ipubi fossil, indicating a greater influence of inorganic processes in its fossilization. Our results also suggest that the Romualdo Formation fossilization process involved the substitution of the hydroxyl group by fluorine, providing durability to the fossils.

## Introduction

Palaemonid crustaceans in Brazil are known from tertiary outcrops, with species occurring in the Tremembé Formation (Oligocene, from São Paulo state) and tertiary deposits in the Marizal Formation (Martins-Neto & Mezzalira, 1991a; Martins-Neto & Mezzalira, 1991b).

The preservation of crustacean fossils is difficult because of their propensity for decomposition (Feldmann & Pole, 1994), what may explain (in part) why only a few species of shrimps are described in Brazilian sedimentary basins. However, the fossils from the Araripe Basin are very well known for their excellent state of preservation and paleobiological diversity (Martill, 1988). On the other hand, the knowledge about fossil shrimp in the Araripe Basin is basically restricted to taxonomic works (Martins-Neto & Mezzalira, 1991a; Martins-Neto & Mezzalira, 1991b; Maisey & Carvalho, 1995; Santana et al., 2013; Pinheiro, Saraiva & Santana, 2014; Saraiva, Pinheiro & Santana, 2018).

The Romualdo and Crato Formations of the Araripe Basin have the following fossil shrimp species: the Crato Formation shows the Carideans *Beurlenia araripensis* (Martins-Neto & Mezzalira, 1991a; Martins-Neto & Mezzalira, 1991b); in the Romualdo Formation were described three species of shrimps, the Sergestoid *Paleomattea deliciosa* (Maisey & Carvalho, 1995), the Penaeoid *Araripenaeus timidus* (Pinheiro, Saraiva & Santana, 2014) and the planktonic shrimp family Luciferidae, *Sume marcosi* (Saraiva, Pinheiro & Santana, 2018).

The two stratigraphic sections of salty water in the Araripe Basin, namely Ipubi and Romualdo Formations, have different paleoenvironments (lagoon and marine, respectively) as well as distinct fossilization processes. The present work employs non-destructive analytic techniques to study two fossilized shrimps and their matrix rocks prospected in these two Cretaceous geological formations of the Araripe Basin. The results obtained allowed us to infer aspects related to the anatomy of the fossils and to the processes of fossilization, diagenesis, and paleoenvironment of crustaceans in the Ipubi and Romualdo Formations.

### Geological setting

The Araripe Sedimentary Basin is one of the most important areas with fossils from the Cretaceous Period in the world, known for the excellent preservation state, diversity and quantity of fossils. This basin is in Northeastern Brazil and comprises part of the states of Ceará, Pernambuco, and Piauí, and it is considered the largest interior Sedimentary Basin of Brazil (Assine, 1992). The most spectacular fossil record is concentrated in the Santana Group, which is composed of the formations Ipubi, Crato, and Romualdo (Valença, Neumann & Mabesoone, 2003).

**Figure 1:**
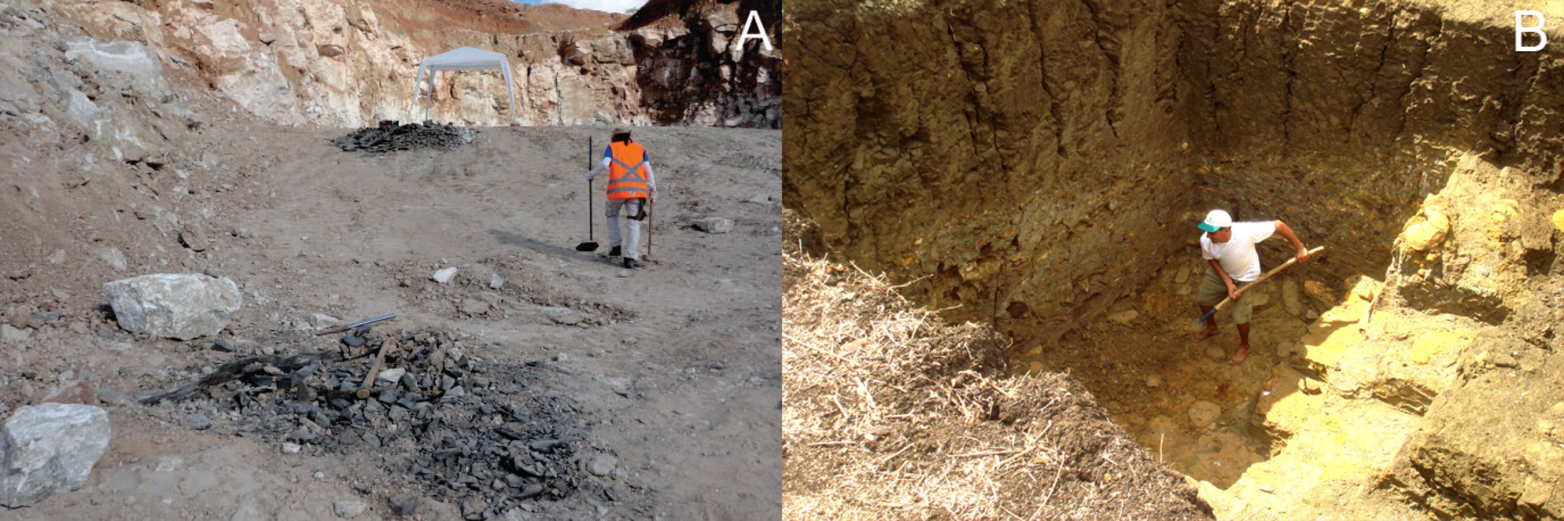
Ipubi and Romualdo excavation sites. (A) Rancharia mining where the specimen from the Ipubi Formation with registered number LPU –918/A was found. (B) Romualdo Formation excavation photo showing the carbonaceous concretions level where was prospected the specimen with registered number LPU - 919. Photos by Olga A. Barros.

The Ipubi Formation (Fig. 1A) represents the evaporitic facies of the Aptian-Albian lagoon system of the basin. It is composed by carbonates with interdigitated shales separated from the Romualdo Formation by a regional unconformity (Cavalcanti & Viana, 1990). The fossils of this unit are found in layers of dark shales, e.g., one of the specimens here studied.

The Romualdo Formation (Fig. 1B) is composed of interbedded shales, marls, and limestones with abundant concretions containing fossils (Valença, Neumann & Mabesoone, 2003). The upper part of the Romualdo Formation consists of layers of calcareous banks with echinoids, bivalve mollusks, ostracods, gastropods and algae mats (Cavalcanti & Viana, 1990). The fossils in these concretions are usually found in large quantity and diversity, e.g., fishes, reptiles, mollusks, crustaceans, palynomorphs, and vegetables (Kellner et al., 2002). The stratigraphic general scheme of the Romualdo Formation is discussed by Pinheiro, Saraiva & Santana (2014).

## Materials and Methods

### Sample study characterization

**Figure 2:**
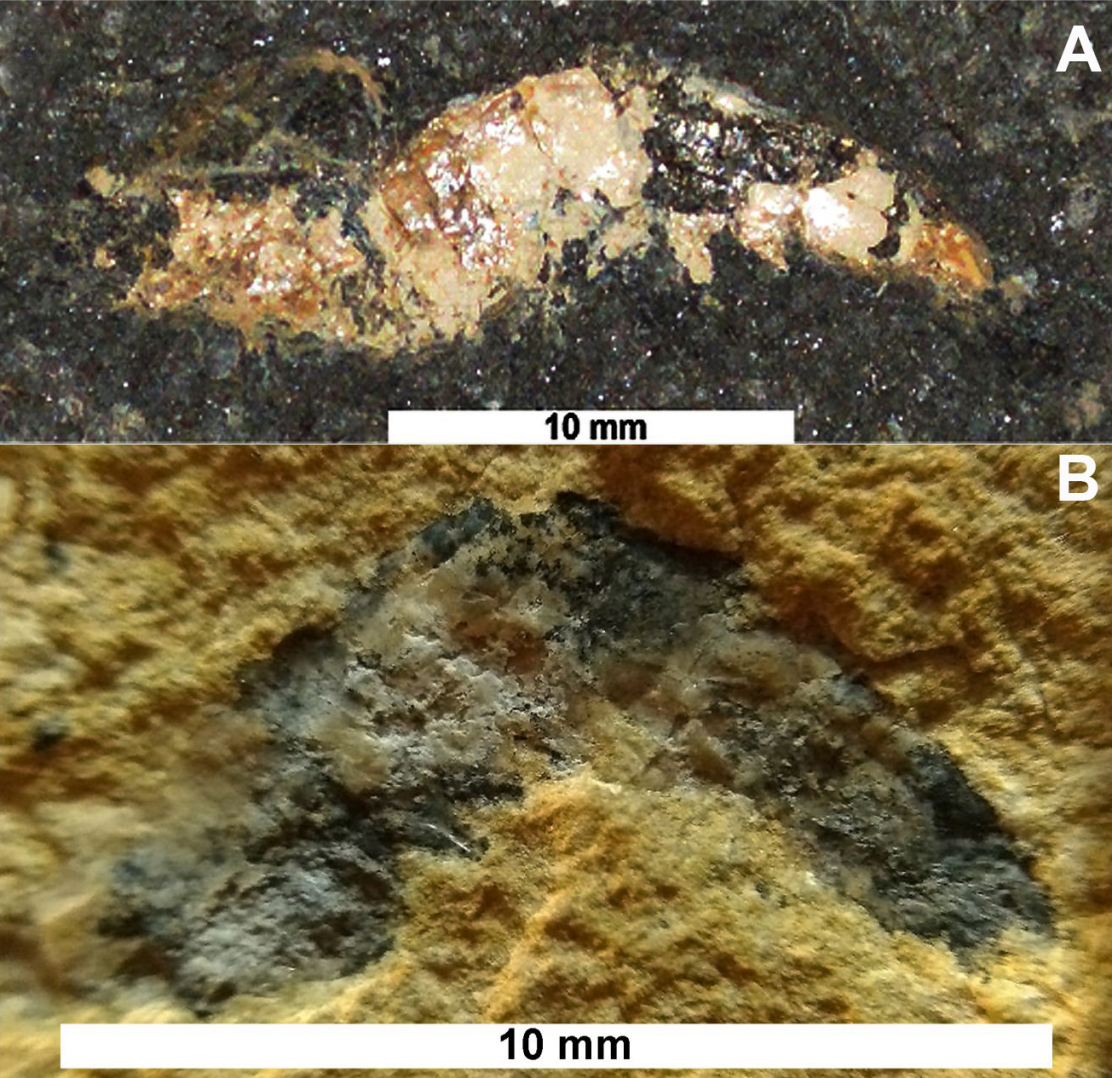
Lateral view of the fossils studied. (A) Optical image of the Ipubi Formation fossil shrimp; (B) optical image of the Romualdo fossil shrimp. Photos by Olga A. Barros.

The materials analyzed consist of two specimens of fossil shrimps (Fig. 2). The first sample (approximately 22 mm of total length, Fig. 2A), LPU - 918/A, prospected in the dark shales of the Ipubi Formation, was collected in Rancharia mining, (7°44′51.1″S/40°28′11.2″W), gypsum exploration area, in the municipality of Araripina—state of Pernambuco/Brazil, in the west portion of the Araripe Basin. An excavation was conducted with the help of a backhoe to extract the dark shales located below the gypsum layer (evaporites).

The second sample (approximately 10 mm of total length, Fig. 2B) collected in the Romualdo Formation, with registered number LPU-919, consists of a carbonate concretion collected in an excavation at the “Parque dos Pterossauros” (Pterosaurs Park) (7°11′32.8″S/39°42′08.5″W) in the municipality of Santana do Cariri-state of Ceará/Brazil. The specimens described here were prepared mechanically and deposited at the Universidade Regional do Cariri (URCA, Brazil).

The fossils and the rock matrices were analyzed by means of vibrational spectroscopy techniques (Raman and Infrared), energy-dispersive X-ray spectroscopy and X-ray diffraction. It is worth mentioning that the present research group had previously studied the EDS/SEM analysis of the same Ipubi Formation shrimp studied here. For further details besides those regarded here, see Oliveira et al. (2015).

### Sample characterization

#### X-ray diffraction (XRD)

The X-ray diffraction patterns were obtained using a Rigaku powder diffractometer with the Bragg–Brentano geometry. The Co–Kα radiation was used and operated at 40 kV and 25 mA. The XRD measurements were taken in the 2θ range from 10° to 100° using step scan procedures (0.02°) in counting times of 5 s. To perform the XRD measurements, 1 g of powder samples extracted from the areas of the fossils and their matrices was used, and for data treatment, the X-pert High Plus score software with powder diffraction files (PDFs) included was used.

#### Infrared spectroscopy

The infrared spectra were measured with powder samples mixed with KBr powder in the proportion 1:100 for each sample (at about 5–10 mg). The pellet thickness varied from 0.5 to 0.6 mm and the acquired spectra were taken in the region from 400 to 4,000 cm^−1^ with a Vertex 70 equipment from Bruker.

#### Raman spectroscopy

The Raman spectra were obtained by a LabRAM HR (Horiba, Kyoto, Japan) spectrometer equipped with a liquid N_2_-cooled CCD detector, 600 l/mm grating using 785 nm laser radiation for excitation.

#### Large-field energy dispersive spectroscopy (EDS) and scanning electronic microscopy (SEM) analysis

Large-field scans were performed in an electron microscope Quanta-450 (FEI) equipped with a GAD (gaseous analytical detector) detector for backscattered electrons (BSE). In addition, an X-ray detector model 150 (Oxford) was used for X-ray energy dispersive spectroscopy (EDS). After the specimens were inserted into the SEM without any preparation, the analyses were performed under low vacuum (approximately 100 Pa for water vapor), by capturing both BSE and X-ray for generating elemental maps. The specimens were scanned at a beam acceleration voltage of 20 kV, with a working distance of 15 mm.

## Results and Discussion

### Description of the specimens under stereoscopic microscope

The sample LPU-919 is a small-sized shrimp (Fig. 2B) excavated in the Romualdo Formation with part of the exoskeleton preserved. Its total length is approximately 10 mm containing carbonaceous concretion from the Romualdo Formation.

The carapace is half preserved, eyes stalk and rostrum, antennae, and antennules are not preserved. The carapace and abdominal pleura are laterally compressed, but it is difficult to distinguish them because the specimen is fragmented.

The abdominal somites are smooth, without spines. It is difficult to observe the pleura of the second somite, however, the sample looks like a Caridea specimen because the pleura of the second somite is heavily rounded at the base. However, the overlapping of the first and the third pleura is not clearly visible because of the specimen preservation state. Pleopods, uropods, and telson were not preserved.

The specimen LPU-918/A is a medium-sized shrimp (Fig. 2A) excavated in the Ipubi Formation with a total length of approximately 22 mm. The antenna and antennule were preserved but is incomplete and curved above of the carapace. Dorsal margin of carapace straight without spines.

The rostrum and scaphocerite are not discernible. The abdomen has six segments, however, the studied specimen looks like the Caridea because the pleura of the second somite is heavily rounded at the base. The same is also observed in the specimen LPU-919.

### Characterization of the fossils

**Figure 3:**
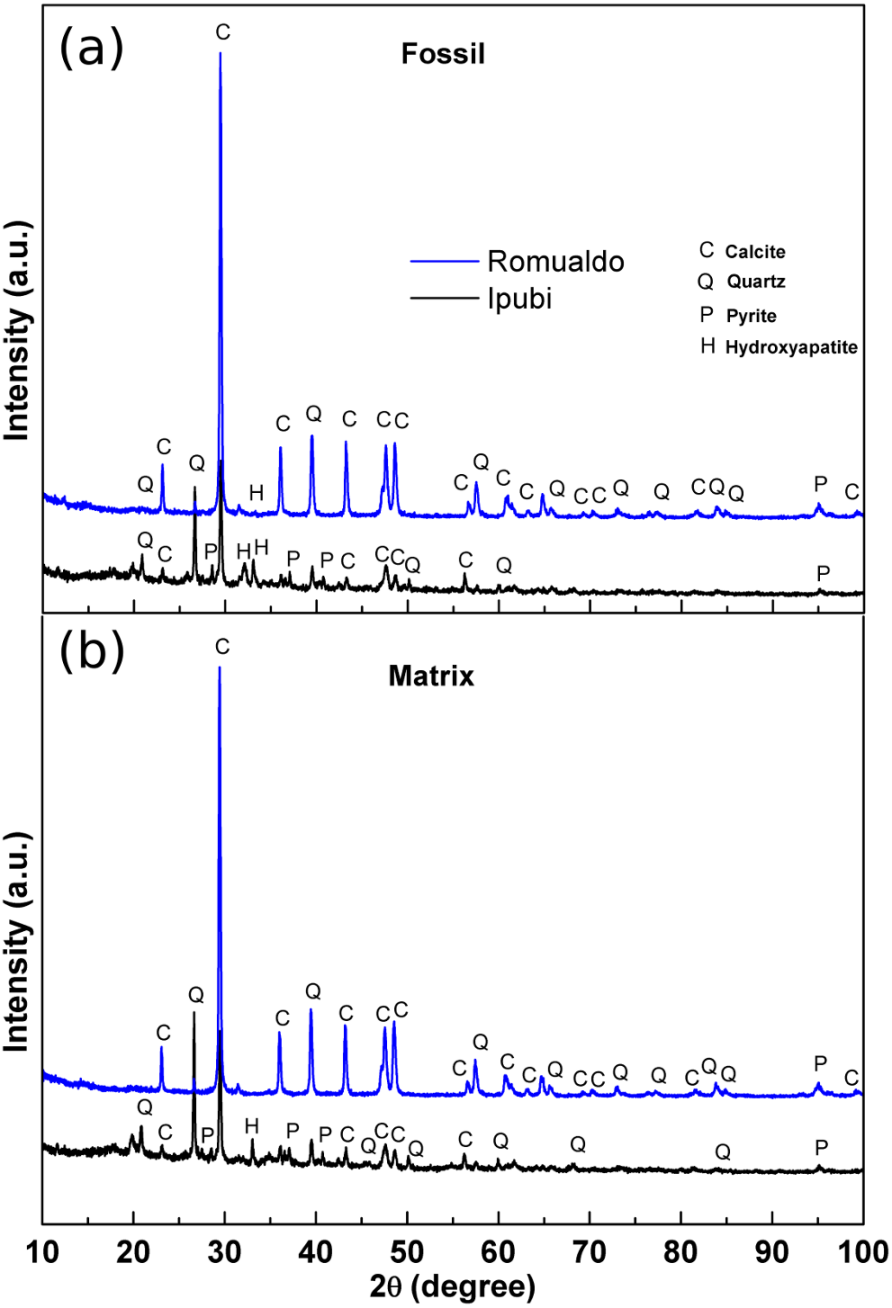
X-ray diffractograms. (A) X-ray diffractograms of the shrimps, (B) X-ray diffractograms of the surrounding matrices. The main phases are indicated as follows: calcite (C), quartz (Q), pyrite (P) and hydroxyapatite (H).

Initial characterization of the shrimp samples was performed by X-ray diffraction measurements, providing structural information about them. The results are shown in Fig. 3 for the fossils as well as for their matrix rocks.

It was possible to identify very significant XRD peaks in the Ipubi Formation fossil shrimp (Fig. 3A), indicating that the major phase is represented by the crystallographic planes belonging to calcite (CaCO_3_). Additionally, other peaks were observed and associated with the crystalline phases of silicon dioxide (SiO_2_, quartz), pyrite (FeS_2_) and hydroxyapatite Ca_5_(PO_4_)_3_(OH). Considering the shrimp sample from the Romualdo Formation, the phases of calcite and quartz seem to be more mixed. It stands out that the hydroxyapatite peak in the Romualdo Formation is less intense than in the Ipubi one, and it shows less phosphate material. For the Ipubi sample, it is also important to observe the presence of several peaks associated with pyrite. It is worth noting that a previous study performed on the Cretaceous specimen of the plant *Brachyphylum castilhoi* from the Ipubi Formation showed the occurrence of pyrite in great quantity, indicating a fossilization mechanism involving FeS_2_ (Sousa Filho et al., 2011).

Osés et al. (2016) considered that the insects from the Crato Formation were pyritized in early diagenesis. Based on this, they proposed that the diffusion of water solutions to and through the insect carcasses, together with their envelopment and infestation by bacteria, created microenvironmental geochemical conditions that led to the mineralization (mainly pyritization) of insect cuticles and internal soft tissues. These geobiological/taphonomic processes have yielded three-dimensional replicas of insects, keeping morphological details of delicate features (e.g., muscle fibers). This can shed light on taxonomy, systematics, and paleoecology.

In our results regarding the X-ray diffraction patterns of the matrices, it is clearly observed that they are quite like the diffraction patterns of the fossil samples. Thus, it can be stated that in the case of the Ipubi Formation matrix there is a calcite dominant phase with a pyrite secondary phase while in the Romualdo Formation the calcite and quartz matrix phases are mixed.

**Figure 4:**
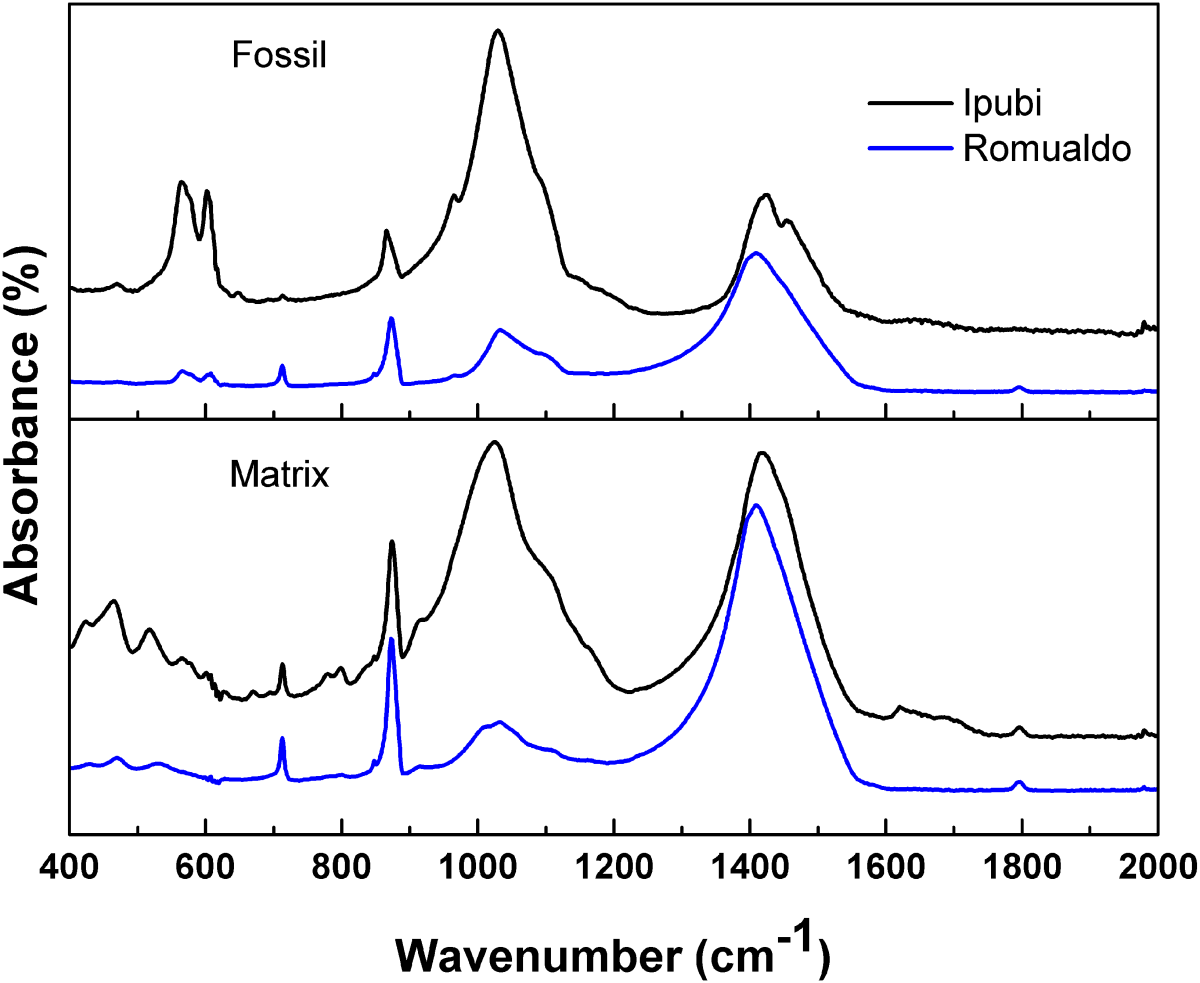
Infrared spectra. Infrared spectra of the shrimps from the Ipubi and Romualdo Formations and their respective matrix rocks.

Figure 4 shows the infrared spectra of the shrimp fossils from both the Ipubi and Romualdo Formations and their respective matrix rocks. The spectra show a good signal-to-noise ratio and they are normalized according to the intensity of the peak at 1,420 cm^−1^. Observing the four IR spectra, the most remarkable peaks are found at 565, 603, 713, 873, 1,030, 1,410, 1,457 and 1,795 cm^−1^, with small wavenumber variations depending on the fossil and the matrix (Fig. 4 and Table 1). The spectra are very similar, in particular, the pair of spectra for the fossil and the matrix from the Romualdo Formation. However, the peaks at 565 and 603 cm^−1^ are only found in the spectra of the fossils. These peaks are assigned to phosphate vibrational modes of hydroxyapatite (Table 1). This fact indicates that hydroxyapatite from the fossils did not migrate to the matrices.

**Table 1:** Wavenumbers of the IR spectra peaks of the fossils. Wavenumbers of the modes appearing in the IR spectra of shrimp fossils and their respective matrices from Ipubi and Romualdo formations. A tentative assignment of the modes is also furnished.

Sample	Ipubi shrimp	Ipubi shrimp matrix	Romualdo shrimp	Romualdo shrimp matrix	Assignment^a^
Wavenumber (cm^−1^)	–	464	–	469	*υ*_4_(PO_4_)
	–	518	–	–	bending Si–O
	–	–	–	532	Si–O–Al bending
	565	–	563	–	*υ*_4_(PO_4_)
	603	–	605	–	*υ*_4_(PO_4_)
		713	713	713	*υ*_4_(CO_3_)
	866				*υ*_2_(CO_3_)
		873	873	873	*υ*_2_(CO_3_)
	–	915	–	–	Al-OH-Al bending
	–	1,023	–	–	Si-O stretching
	1,030	–	1,035	1,035	Si-O stretching
	–	–	1,410	1,410	*υ*_3_(CO_3_)
	1,422	1,420	–	–	*υ*_3_(CO_3_)
	1,457	–	–	–	*υ*_3_(CO_3_)
	–	1,795	–	1,795	(*υ*_1_+ *υ*_4_)(CO_3_)

**Notes.**

a References (Gunasekaran, Anbalagan & Pandi (2006), Legodi & Waal (2007), Silva et al. (2013b), Silva et al. (2013a), Alencar et al. (2015) and Sousa Filho et al. (2016)).

The main differences in the IR spectra of the two fossils are observed in the relative intensities of the peaks around 1,410 cm^−1^ (*υ*_3_(CO_3_)) and 1,030 cm^−1^ (Si-O stretching). Thus, the Romualdo Formation fossil showed a greater quantity of calcite than the Ipubi one. However, considering the results in a broader way, the IR spectra allow conjecturing that the Ipubi and Romualdo Formations processes have similar IR-active chemical structures.

The infrared spectra of the Ipubi Formation fossil shrimp presented vibrational bands associated to the phosphate group, the main constituent of hydroxyapatite (Ca_10_(PO_4_)_6_(OH)_2_), in agreement with the EDS results of Oliveira et al. (2015). These authors studied the same Ipubi sample considered here (LPU - 918/A) and, according to them, the major phase was formed by calcite with pyrite and traces of zinc sulfide in some specific points of the fossil. The XRD and IR results obtained in the present work agree with the results of Oliveira et al. (2015).

**Figure 5:**
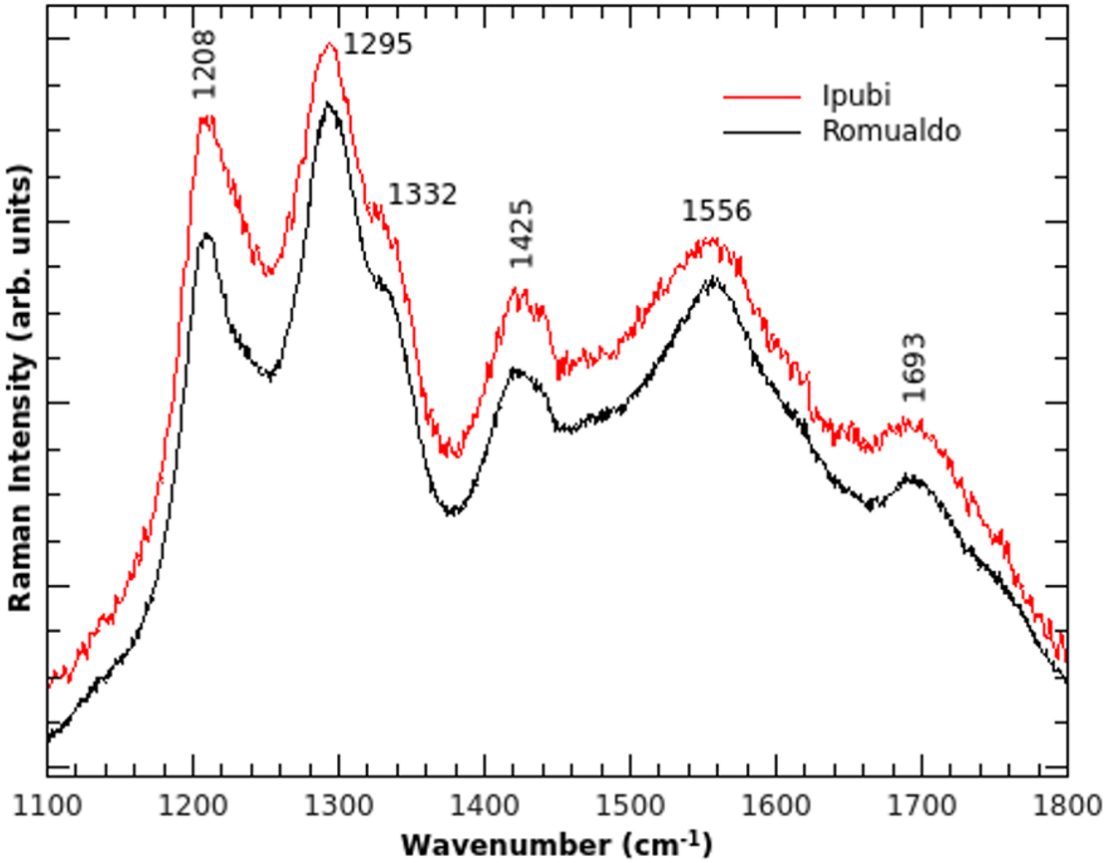
Raman spectra. Raman spectra of the shrimp fossils from the Ipubi and Romualdo Formations.

Further analysis was carried out with Raman spectroscopy (Fig. 5), which can furnish interesting insights about the vibrational properties of the material. Raman spectroscopy can corroborate the data obtained through IR spectroscopy and, in some cases, can give complementary information (Oliveira et al., 2015). Interestingly, the Raman spectra are very similar regardless of the geological formation. This might suggest that there is a common point in the fossilization processes. One possible interpretation might be related to the decomposition process involving original material common to both fossils, for example, cuticles.

Preservation of the soft tissues was mentioned in fish (Wilby & Martill, 1992), pterosaurs (Kellner, 1996), theropod (Kellner, 1996) and insects (Osés et al., 2016) from the Araripe Basin. Delgado et al. (2014) performed similar analyses in insects from the Crato Formation and observed that pyrite has replaced the soft tissues of microstructures of the internal cavities (cuticles). For Osés et al. (2016), insects from the Crato Formation are the first record of these organisms in lacustrine laminated limestones preserved by pyrite without a volcanogenic sedimentary origin. However, for the Crato Formation, sulfate was likely provided by evaporites. Anyway, despite commonly preserved in continental setting limestones fossil insects are rarely pyritized given the scarcity of sulfate available in such depositional environments (Martínez-Delclòs, Briggs & Peñalver, 2004).

In our results it was still possible to identify that the samples of both formations had calcite in their majority phases, confirming the results here sampled by XRD, Infrared and Raman. A large amount of CaCO_3_ found comes from its precipitation around the fossiliferous material.

For Martill (1988), despite that there was a large amount of calcium carbonate (CaCO_3_) precipitated around the fossiliferous material, only soft tissue preservation is related to phosphatization. This type of preservation by authigenic apatite or hydroxyapatite often allows preservation with high fidelity of morphological details (Martill, 1990; Wilby, 1993).

According to the results of Briggs & Kear (1994), in spite of the precipitation of calcium carbonate around the carapace of fossilized shrimps, only calcium phosphate replaced the preserved nonresistant tissues. This is similar to what happens to some fishes from the Araripe Basin. Briggs & Kear (1994) results pointed to a phosphatized diagenetic mineralization.

Mineralization of nonresistant tissues by calcium phosphate is very sensitive to external variables. Experiments in anoxic environments considering aerobic and anaerobic decomposition found that there was no difference between decomposition rates, but the anoxic condition of the medium was important, inhibiting the processes of necrophagy and bioturbation. Such a fact facilitates the preservation of hard and articulated parts, even when there is no preservation of non-resistant tissues (Allison & Briggs, 1991). The special conditions of temperature, pressure, and pH influence the phosphatization of these tissues (Allison & Briggs, 1991; Briggs & Kear, 1994).

According to Martill (1988), vertebrate fossils of the Romualdo Formation consist predominantly of calcium carbonate (CaCO_3_) together with minor amounts (at a lower concentration) of Ca_10_(PO_4_)_6_(OH)_2_ (hydroxyapatite). This was subsequently confirmed by XRD analysis and infrared spectroscopy by Lima et al. (2007) for a fish fossil. Additionally, Schultze (1989) discovered that fish muscle tissues were preserved by calcite (CaCO_3_) in crystalline form, based on chemical analysis by X-ray diffraction (XRD) measurements performed in the tissue.

Considering the discussion above, the Raman modes can be assigned as follows. The peak observed at 1,425 cm^−1^ corresponds to the *υ*_3_(CO_3_) vibrational mode and it is also found in the IR spectra. The Raman band at 1,332 cm^−1^ is assigned to the D-band (Ferrari & Robertson, 2001), which is commonly found in carbon materials with sp^3^ hybridization, and it is related to structural disorder in carbonaceous materials. The G-band of carbon can be assigned to the shoulder observed in 1,621 cm^−1^. The vibrational modes at 1,208, 1,295, 1,556 and 1,693 cm^−1^, consequently, are tentatively assigned to the stretching vibration *υ* (CC) from carbon structures. This strengthens the hypothesis that carbon is the result of the decomposition of the original organic matter because no Raman peak was identified in the matrices of both fossil samples.

**Figure 6:**
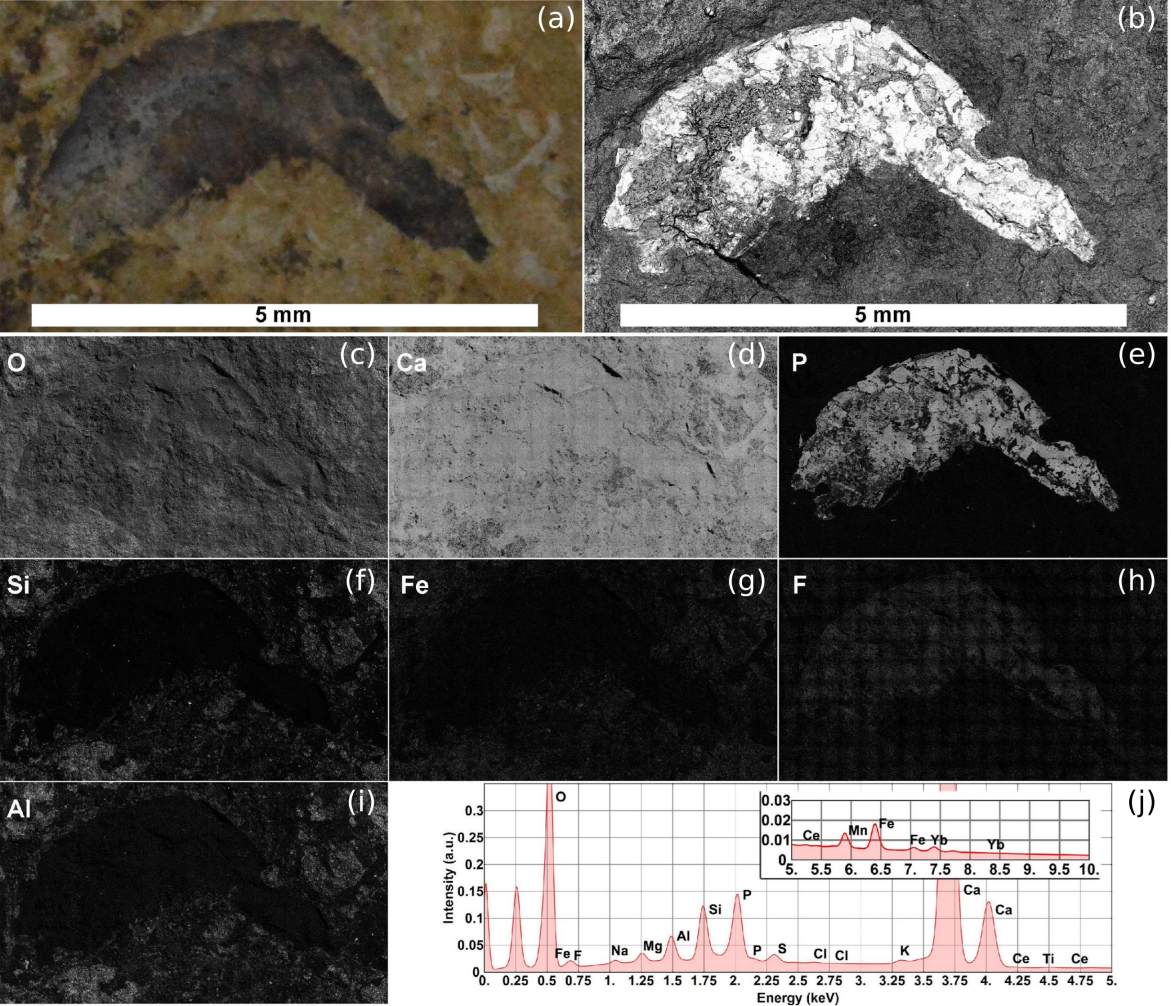
SEM image and EDS elemental maps of the Romualdo Formation sample. (A) Optical image of the Romualdo Formation shrimp fossil. (B) Electron micrograph of backscattered electrons (BSES) of the Romualdo shrimp fossil. (C–I) EDS elemental maps shown from highest (C) to lowest (I) relative weight concentration (wt%) calculated in accordance with the spectrum obtained by the wide-field scan. (J) Full spectrum of the entire area of the fossil. Photos by Naiara C. Oliveira.

The analysis of the fossil samples was also performed using scanning electron microscopy and energy-dispersive X-ray spectroscopy. The fossil material analyzed has a size of about 12 mm in length and 7 mm in width (Figs. 6A and 6B). For the image acquisition of the whole fossilized area in high definition, a large-field scan was performed in which the microscope acquires thousands of images with a suitable magnification covering the sample area. Throughout the interaction of the electron beam with the sample, two signals were collected simultaneously: the backscattered electrons (BSE) and the emitted X-ray. The BSEs provide a contrast function on images related to the atomic number Z of the elements that constitute the sample (BSE Z, Fig. 6B). This allows further elucidation of the morphology of specimens compared with the images from optical microscopy (Fig. 6A).

The X-rays emitted during the analysis provide information about the chemical composition of the samples. EDS is a spectroscopic technique of great value for samples with high heterogeneity in their compositions since it can detect the presence of major and minor chemical elements. (Figures 6C–6I) shows only elemental maps of the Romualdo Formation sample with relative mass concentration (wt%) over 1.0%. Table 2 gives a relative quantification of the chemical elements detected in the Romualdo Formation shrimp fossil. Note the predominance of the elements calcium and oxygen the whole fossil material area, i.e., fossil and matrix, in accordance with the calcium carbonate composition. The large-field images were generated by assembly of about 1,020 adjacent images obtained during the entire scan of the sample at 1,000× magnification. Figure 6J shows the total EDS spectrum of the Romualdo Formation shrimp fossil with the cumulative sum of all spectra (1,020 fields) obtained during the whole analysis.

**Table 2:** Relative quantification of the chemical element present. Relative quantification of the chemical elements detected by wide field scan on the shrimp fossil from Romualdo Formation. The values correspond to relative values calculated from the software Aztec (standardless method) given the concentration of the chemical elements; the correlation and normalization is given by the intensity of the peaks corresponding to all chemical elements detected.

Elements	Atomic weight (g mol^−1^)	Relative %- weight (wt%)
O	15.99	49.0
Ca	40.07	39.3
P	30.97	3.1
Si	28.08	2.2
Fe	55.84	1.5
F	18.99	1.5
Al	26.98	1.1
Mn	54.93	0.8
Mg	24.30	0.5
S	32.06	0.4

**Figure 7:**
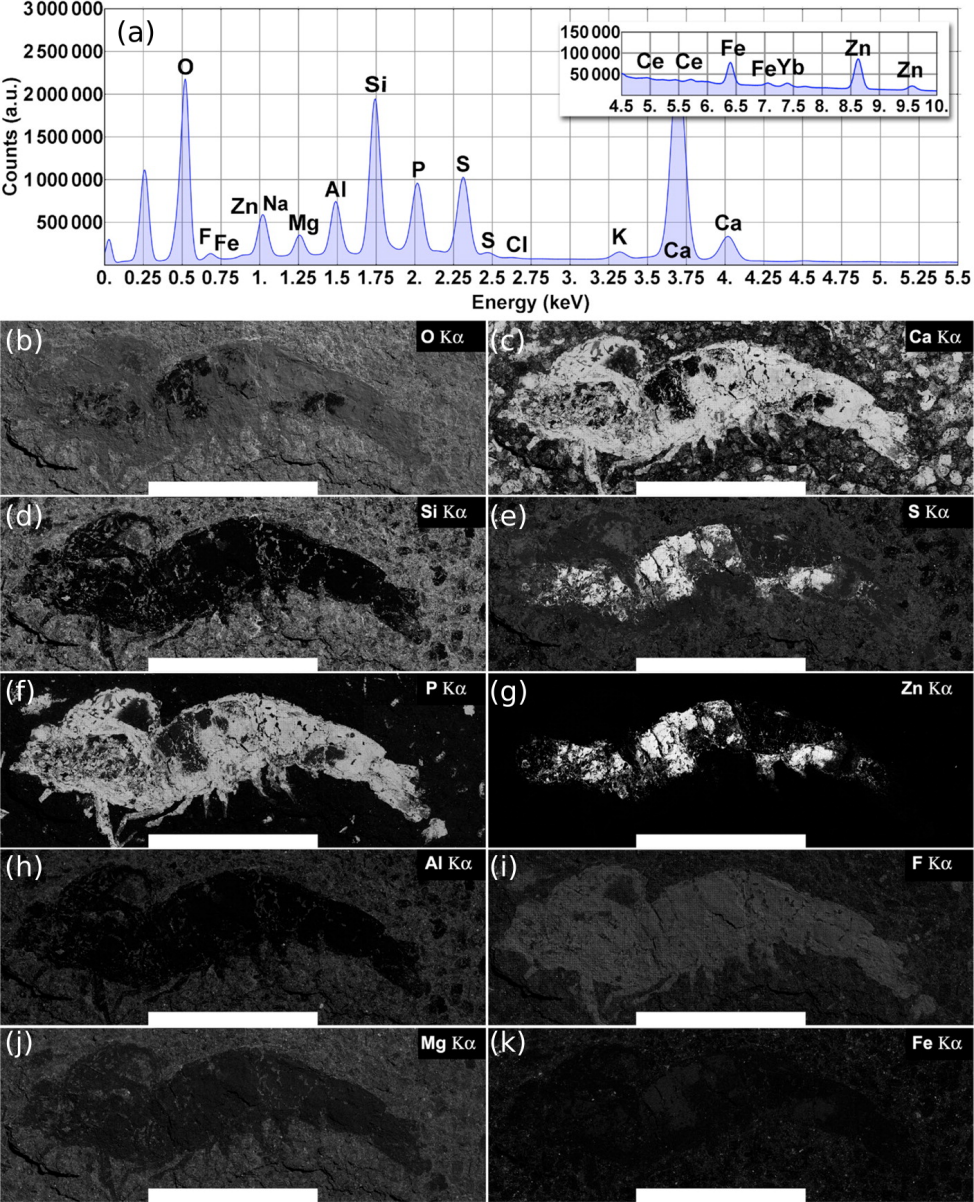
Ipubi fossil shrimp EDS images. (A) Total EDS spectrum for the whole fossil area. (B–K) Elemental maps are represented from left to right, top to bottom, as a function of their relative concentrations (wt %) calculated from the total energy-dispersive X-ray spectrum. Elements with quantities larger than 1.0 wt % are shown. The white scale bar corresponds to 10 mm. Reprinted with permission from Oliveira et al. (2015). Copyright (2015) American Chemical Society.

On the other hand, the total EDS spectrum (3,600 fields) obtained through the large-field scan for the whole fossil shrimp area previously studied from the Ipubi Formation (Oliveira et al., 2015) is represented in the top panel of Fig. 7. Large-field images with compositional information of the specimen are represented in the bottom panels of the same figure. As shown in Fig. 7, the correlation between elemental maps of the phosphorus and calcium mineralized in the fossil as hydroxyapatite (Ca_5_(PO_4_)_3_OH) makes it possible to identify its morphologic features such as pleopods, pereopods, antennae, antennules, and somites.

According to Oliveira et al. (2015), it was observed that the Ipubi Formation fossil shrimp presented three regions with characteristic elemental composition: the matrix which contains mainly O, Si, Ca, S, Al, Mg, and Fe (region A), a phosphorus-rich region of the fossil constituted of O, Ca, P, F, S, and Na (region B), and finally a region in the fossil containing mostly S and Zn (region C), see Table 3. The correlation of large field elemental maps with EDS spectra of specific regions of the fossil allowed to identify crucial aspects of its fossilization processes. As reported by Oliveira et al. (2015), along with the fossilization of the animal through the formation of hydroxyapatite (Ca_5_(PO_4_)_3_OH), zinc sulfide (ZnS) was also formed, interfacing with the hydroxyapatite. Furthermore, the presence of the element fluorine in the phosphorus-rich region indicates a possible formation of fluorapatite (Ca_5_(PO_4_)_3_F).

**Table 3:** Ipubi fossil EDS. Relative quantification of the elements detected in the Ipubi Formation fossil shrimp. Adapted with permission from Oliveira et al. (2015). Copyright (2015) American Chemical Society.

			Scanning at specific regions^b^		
		Cumulative: LF scanning^a^	A	B	C
Element	Atomic mass (g mol^−1^)	wt%^c^	wt%^c^	wt%^c^	wt%^c^
O	15.99	42.9	48.9	40.3	6.5
F	18.99	2.3^d^	^e^	5.6	0.2^d^
Na	22.99	0.6^d^	^e^	1.6^d^	^e^
Mg	24.30	1.6^d^	2.1^d^	0.4^d^	0.3^d^
Al	26.98	3.5^d^	5.1	0.5^d^	0.6^d^
Si	28.08	9.4	14.8	1.3^d^	1.2^d^
P	30.97	5.2	0.2^d^	11.7	0.8^d^
S	32.06	5.9	6.2	3.2^d^	28.0
Cl	35.45	0.1^d^	^e^	0.2^d^	^e^
K	39.09	0.7^d^	1.2^d^	0.1^d^	0.4^d^
Ca	40.07	21.5	18.6	33.6	3.1^d^
Ti	47.86	0.1^d^	0.3^d^	^e^	^e^
Fe	55.84	1.3^d^	2.6^d^	0.2^d^	0.5^d^
Zn	65.38	4.5	^e^	1.3^d^	58.4
Ce	140.12	0.1^d^	^e^	^e^	^e^
Yb	173.05	0.3^d^	^e^	^e^	^e^

**Notes.**

a Values calculated fgom the total EDS spectgum for the whole fossil area.

b Values calculated from the EDS spectra from specific regions of the fossil.

c %-Weight values are relative values obtained after the calculation of the element concentgation by the software (standardless method), and after the correlation and normalization of all peaks intensities.

d %-Weight values fog elements present in low concentrations could not be quantitatively interpreted due to the precision of the EDS method used, although they could be precisely detected.

e Trace elements were detected through the cumulative spectrum generated along the large-field scan. These trace elements could not be detected in single spectra.

The amount of the element phosphorus found by EDS analysis in the Romualdo Formation fossil shrimp was quite sparse compared to the results found for the Ipubi fossil (Oliveira et al., 2015), confirming what was found by the other techniques employed in this work. The presence of fluorine and the observation that its EDS map overlaps with the one of phosphorous can provide additional information about the fossilization process (Figs. 6E and 6H). There was possibly a partial replacement of the hydroxyl group of hydroxyapatite (Ca_5_(PO_4_)_3_OH) by fluorine. This substitution might favor the formation of fluorapatite (Ca_5_(PO_4_)_3_F). The ionic radii of (OH) and F are similar, while F is easily found in pegmatitic/granitic rocks that are very abundant in the state of Ceará (Brazil). Fluorapatite is physically more stable and could provide greater durability to the fossil.

Figure 7 shows the EDS elemental map for the Ipubi sample. This figure is reprinted with permission from Oliveira et al. (2015) who worked with the same Ipubi sample. Copyright (2015) American Chemical Society. Compared with the Romualdo Formation fossil, it was observed that the Ipubi one has better preservation of fossilized materials and better visibility of anatomical details (abdominal somites, uropods, and telson). It was also observed that part of the Ipubi fossil material was further replaced in a minor phase of silicon, pyrite and zinc sulfide. Both samples had calcite formations in their major phases, in agreement with the Infrared and Raman results.

## Conclusions

Complementary experimental techniques were employed to investigate fossils from the Romualdo and Ipubi Formations. We concluded that in both fossil samples, the fossilization process occurred mainly through substitution by calcium carbonate (majority phase). This is reinforced by the fact that the IR spectra of both fossils are similar. The same can be stated about the matrices. The similarities are also present in the XRD diffractograms. Ipubi shrimp, however, showed a greater number of pyrite peaks in the XRD diffractogram.

For the Ipubi Formation sample, part of the fossil material was further replaced in a minor phase of silicon, pyrite and zinc sulfide, confirming previous results. After the death of the individual, there was loss of organic material in some specific points of the shrimp, suggesting that these regions might be more susceptible to the decomposition followed by material replacement. The Ipubi fossil is better preserved than the Romualdo one, possibly because the first has traces of pyrite and phosphate.

The Romualdo Formation shrimp preserved in less detail presented a greater amount of calcium carbonate (CaCO_3_) and a greater substitution of the original hydroxyapatite. Probably this specimen was submitted to greater environmental stress (carriage) between the place of death and the final deposition site, or it received the greater influence of the diagenesis, hindering the visualization of morphological details. Importantly, there was possibly a partial replacement of the hydroxyl group of hydroxyapatite (Ca_5_(PO_4_)_3_OH) by fluorine in the fossilization process of the Romualdo Formation fossil.

Environmental variations in pH, temperature, and pressure might have influenced the solubility of some compounds. The fossil from the Romualdo Formation was replaced predominantly by calcium carbonate (CaCO_3_) originated from precipitation in the aqueous medium. It could have also occurred diagenetic recrystallization that prevented the preservation of morphological details such as the divisions of the abdominal somites, uropods, and telson. Structures of the pereiopods and pleopods, antennas and antennules were not visualized in the sample, likely because they are anatomic structures more sensitive to decomposition. Finally, the involvement of these quantitative techniques contributes to obtaining a range of additional information that goes beyond the analysis of the fossil itself; it is possible, for example, to access the taphonomic history of organisms, providing a more reliable view of ancient life.

## Additional Information and Declarations

### Data Availability

The following information was supplied regarding data availability: Barros, Olga (2018): Fossil Shrimp. figshare. Fileset. https://figshare.com/articles/Fossil_Shirmp/7250063.
